# *Nocardia arthritidis* scleritis: A case report

**DOI:** 10.1016/j.ajoc.2023.101794

**Published:** 2023-01-12

**Authors:** Omar Abdelmegid, Shambhawi Thakur, Albert Y. Cheung

**Affiliations:** a University of Massachusetts Medical School, Department of Ophthalmology, Worcester, MA, USA; b Eastern Virginia Medical School, Department of Ophthalmology, Norfolk, VA, USA; c Virginia Eye Consultants/CVP, Norfolk, VA, USA

**Keywords:** Infectious scleritis, Nocardia, Scleritis, Nocardia arthritidis, OD, Right eye, OS, Left eye, HIV, Human Immunodeficiency Virus, LASIK, laser-assisted in-situ keratomileusis, TMP/SMX, trimethoprim-sulfamethoxazole, BCL, bandage contact lens

## Abstract

**Purpose:**

This report describes a case and management of a 69-year-old female with infectious scleritis found to be caused by *Nocardia arthritidis* species.

**Observations:**

Our patient presented with severe constant pain in the left eye (OS) following cataract surgery. She had a pertinent past medical history significant for renal transplantation (on oral tacrolimus, mycophenolate, and prednisone). Slit lamp examination OS (1 month after cataract surgery) demonstrated 3+ injection temporally accompanied by scleral thickening and multiloculated abscesses with purulent drainage from small conjunctival erosions. The abscesses were debrided and sent for gram stain and culture. The patient was treated with repeated subconjunctival injections of antibiotics and an antifungal; topical amphotericin, vancomycin, and amikacin; and oral trimethoprim-sulfamethoxazole (double strength). Two separate gram stains with cultures confirmed the diagnosis and species identification. The patient responded well to repeat subconjunctival injections early on in addition to the prescribed regimen, remaining free of disease at the last follow-up (12 months following presentation).

**Conclusions and Importance:**

This unique case demonstrates infectious scleritis caused by an uncommon Nocardia species (*N. arthritidis*) that was successfully treated with similar strategies used for other reported Nocardia species. As Nocardia scleritis can lead to adverse outcomes if not treated promptly and properly, it should be considered on the differential diagnoses in an immunocompromised patient who presents with acute ocular symptoms after any recent ocular surgery.

## Highlights

• *Nocardia arthritidis* is an uncommon cause of Nocardia scleritis.
• The symptoms include unilateral ocular pain, redness, and swelling.
• Our case responded to a combination of topical, oral, and injection antibiotics.
• If not treated promptly, it could cause detrimental impact on vision.

## 1 Introduction

Nocardia is a genus of saprophytic bacteria that can be found within soil rich in organic matter. Its species is usually present in oral flora, but not in the eye or respiratory tract, and it can be introduced by trauma, surgery, or through inhalation.^1^ Nocardia species rarely cause infection due to its low virulence. Thus, infections caused by Nocardia species often manifest as opportunistic infections in immunocompromised patients, organ transplant recipients, and patients infected with Human Immunodeficiency Virus (HIV).^1^

Nocardia species often have subtle presentations and are usually challenging to diagnose as it is not commonly encountered in clinical practice and can mimic other etiologies. It is recognized to have more than 85 species. Although uncommon, complications from Nocardia infection can be devastating to the eye. Ocular sight-threatening manifestations include scleritis, keratitis, and endophthalmitis.^1^ Herein, we report a unique case of *Nocardia arthritidis*, a rare Nocardia species, as an isolated etiology of scleritis. *N. arthritidis* is considered to be one of the least encountered forms of Nocardia species with this presentation.

## 2 Case report

A 69-year-old female with a past ocular history of glaucoma, dry eye syndrome, laser-assisted in-situ keratomileusis (LASIK), and recent cataract surgery (1 month prior) was referred for severe constant pain in the left eye (OS). Additionally, she had a past medical history significant for renal transplantation (on oral tacrolimus, mycophenolate, and prednisone), elevated cholesterol, and depression. Symptoms began 1 week after her cataract surgery without any overt eye trauma. At that time, the surgeon had noted temporal conjunctival/scleral injection that had slowly worsened over the next couple weeks, despite starting oral ciprofloxacin, ofloxacin ophthalmic solution every 2 h, and difluprednate twice daily. Initial cultures (swab of conjunctiva/sclera) had been taken but had not yet yielded any results. She had also been continued on timolol OS and latanoprost in the right eye (OD).

**Fig. 1:**
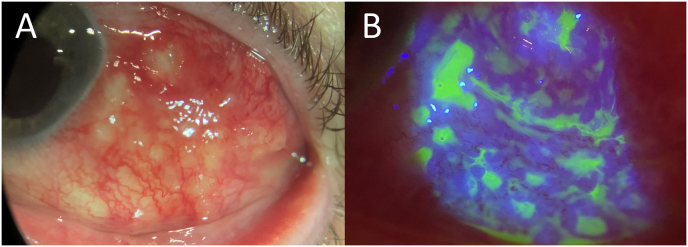
Slit lamp photograph at presentation demonstrates left eye conjunctival/scleral injection temporally accompanied by multiloculated abscesses with purulent drainage (1A). Fluorescein demonstrates multiple small conjunctival erosions in the area (1B).

Our slit lamp examination OS (1 month after cataract surgery) demonstrated 3+ injection temporally accompanied by scleral thickening and multiloculated abscesses with purulent drainage from small conjunctival erosions (Fig. 1A and B). There was no corneal involvement with a sealed clear corneal cataract incision and intact LASIK flap. The involved area was peripheral/posterior to the pre-existing cataract incision. Remainder of the slit lamp and dilated fundus examinations were unremarkable. The abscesses were debrided and sent for repeat gram stain in addition to bacterial and fungal cultures. Both a swab and scrapings (plated on blood, chocolate, and Sabouraud agars) from the conjunctiva/sclera were sent. Subconjunctival injections of vancomycin, ceftazidime, and amphotericin were administered. Diagnostic lab testing was also obtained for infectious and autoimmune etiologies, including antinuclear antibodies, angiotensin-converting enzyme, anti-neutrophilic cytoplasmic autoantibody, complete blood count with differential, erythrocyte sedimentation rate (ESR), fluorescent treponemal antibody absorption test, Human Leukocyte Antigen B27, Lyme disease antibodies, lysozyme, QuantiFERON TB gold test, and rheumatoid factor.

**Fig. 2:**
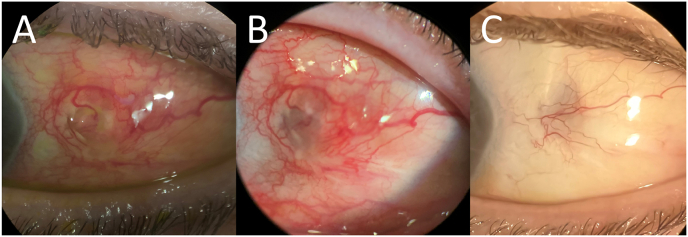
Slit lamp photograph demonstrating improvement (smaller conjunctival defects/staining, less injection) 3 weeks after debridement, subconjunctival injections, and change in therapy (2A). By 4–5 weeks, there was resolution of the conjunctival defects, less injection, conjunctival scarring, and scleromalacia (2B). At 6 months follow-up, slit lamp photograph demonstrates a white, quiet eye with mild scleromalacia (2C).

The initial management plan consisted of topical amphotericin, vancomycin, and amikacin every 2 h, cyclopentolate 1% three times a day, and oral trimethoprim-sulfamethoxazole (TMP/SMX, double strength) 800 mg–160 mg twice a day as the initial gram stain obtained by the referring physician demonstrated gram-positive, branching rods. There was a positive response to therapy, so repeat subconjunctival injections of vancomycin, gentamicin, and ceftazidime were administered 3 days later. The cytology demonstrated a few epithelial cells and many white blood cells. Acid-fast bacilli were identified on the smear; no yeasts or hyphal elements were identified, so we recommended discontinuation of the anti-fungal therapy. Injections were repeated 3 days following this (gentamicin was replaced by amikacin) and 1 week after that. There was gradual closure of the conjunctival defects, disappearance of the abscesses, and improvement in the temporal inflammation (Fig. 2A&B). The topical antibiotics were decreased to 4 times daily after closure of the conjunctival defect.

**Fig. 3:**
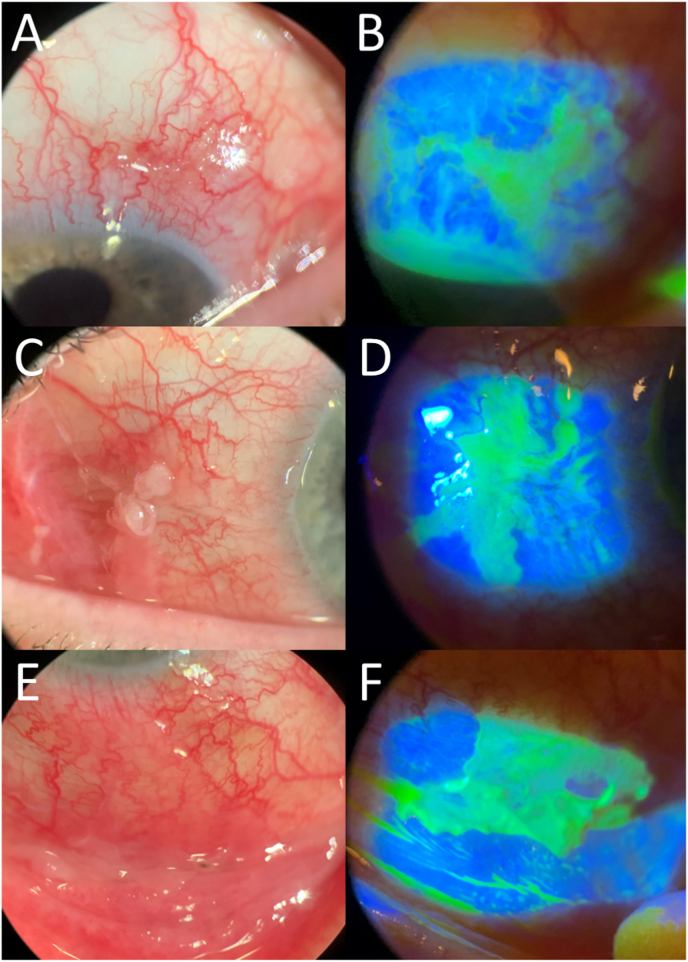
Slit lamp photographs (with and without fluorescein) depict examples of the conjunctival defects with a serpentine border along with mild fibrosis and pseudomembranes, affecting the superior (3A and 3B), nasal (3C and 3D), and inferior (3E and 3F) conjunctiva.

At the one-month follow-up, the culture specimens were determined to be *Nocardia arthritidis*. At the 5-week follow-up, the patient noted itchiness, redness, and yellow discharge in the morning OS with no associated pain. Upon slit lamp examination, localized 1–2+ conjunctival injection and a temporal conjunctival defect were observed. The conjunctival defect had a serpentine border along with mild fibrosis and pseudomembranes (Fig. 3). The additional symptoms and slit lamp findings prompted administration of a gentamicin subconjunctival injection to the area and increased frequency of the topical vancomycin and amikacin back to every 2 h OS. Valacyclovir was also started 500 mg three times daily to cover any potential Herpes Simplex Virus-related disease in the setting of conjunctival defects with irregular (potentially pseudodendritic) borders and immunosuppression. Culture and gram stain of the conjunctival defect were negative. The conjunctival defect appeared to heal following the injection, but the appearance of a new conjunctival defect in another separate area occurred (Fig. 3). Repeat subconjunctival amikacin (or gentamicin) ± vancomycin injections were administered weekly in the OS for the following 5 weeks for cyclical defects appearing in different locations. Similarly, each conjunctival defect responded to a localized subconjunctival antibiotic injection. After the patient had difficulty tolerating the oral TMP/SMX, doxycycline was started 100 mg twice daily after discussion with our Infectious Disease service.

Likely due to underlying neurotrophic keratitis (secondary to prior LASIK) exacerbated by drop toxicity, the patient experienced a corneal epithelial defect and increased superficial punctate keratitis that responded well to bandage contact lens (BCL) therapy and decreased antibiotic drop frequency (4–6 times daily). After another conjunctival defect appeared, the decision was made to biopsy this lesion to exclude any concurrent infectious etiology. This demonstrated chronic conjunctival inflammation and scarring with associated amorphous acellular material in the substantia propria. No organisms were seen on the conjunctival biopsy, and the special stains (acid-fast, fungal, and gram) were all negative.

Fortunately, there were no further conjunctival defects by 4 months of treatment. While still on topical amikacin, a brief course of topical prednisolone was used to stabilize the eye after the biopsy returned negative for any organisms.

At this point, the patient discontinued doxycycline due to intolerance to the medication with associated vomiting episodes. At the six-month follow-up, the conjunctiva appeared quiet with no signs of infection (Fig. 2C). Topical antibiotics were discontinued as the Infectious Disease department recommended a minimum of 6 months of treatment. Oral therapy had been continued for approximately 4 months; however, there was a period after 3 months when she could not take the TMP/SMX consistently due to intolerance. She has remained free of disease 12 months after the initial presentation.

## 3 Discussion

Nocardia is not a part of the normal flora of the eye, and hence, can cause opportunistic infections if introduced through ocular trauma or surgery in immunocompromised patients.^1^

*N. arthritidis*, like other Nocardia species, is a strictly aerobic, gram-positive, acid-fast, rod-shaped bacillus.^2^^,^^3^ It was first isolated in 2004 from the sputum and inflammatory exudates of a rheumatoid arthritis patient.^4^ Later, it was found to be involved in other systemic infections. For example, it was observed in brain lesions of a patient with past medical history of silicosis and was found to be the cause of an infection in a retrotracheal necrotic mass in another immunocompromised patient.^5^^,^^6^ Since its discovery in 2004, *N. arthritidis* and *N. asteroides* have been considered to be important etiologies of ocular nocardiosis. In one study, *N. asteroides* was associated with 3 cases of keratitis, and in another study in Nepal, *N. arthritidis* was reported to be found in a case of keratitis.^4^^,^^7^ *N. asteroides* has been reported in the literature as a common cause of scleritis, but to date, no reported case has documented scleritis caused by *N. arthritidis*.^1^^,^^8^ As our case was referred by an outside ophthalmologist, there were two separate cultures that confirmed the species.

Nocardia scleritis clinically manifests with intense ocular pain, photophobia, blepharospasm, and eyelid swelling.^9^ Its clinical signs include scleral thinning, necrotic areas, hemorrhage, discharge, scleral abscesses, dilated episcleral vessels, and conjunctival and scleral inflammation. The presented case exhibited similar signs and symptoms as reported for scleritis caused by other Nocardia species. Interestingly, there have been mixed theories about whether Nocardia scleritis occurs as a primary infection or as an extension of a pre-existing corneal infection to the limbus.^1^^,^^10^^,^^11^ Our case did not demonstrate any signs of concomitant keratitis.

Since Nocardia scleritis is rare and may present with subtle symptoms, it can be difficult to detect. Additionally, the clinical presentation can mimic scleritis that resembles other causative organisms or processes. Studies show that Nocardia and fungi may be the most common microbial agents that cause scleritis in developing countries, but bacteria, especially pseudomonas, are most common elsewhere.^12^^,^^13^ It is critical to differentiate between Nocardia and fungal scleritis as both can have similar risk factors and clinical presentations yet very different treatment regimens.^1^ In the presented case, the initial treatment regimen included an antifungal drug which was later discontinued when gram-positive rods were isolated from the gram stain/culture. Moreover, other gram-positive bacteria like *Moraxella catarrhalis*, non-tuberculosis mycobacterium, *Corynebacterium diphtheriae*, *Gordona rhodococcus*, and *Tsukamurella* can exhibit signs and symptoms indistinguishable from Nocardia scleritis.^9^^,^^12^ Similarly, metabolic disorders, autoimmune diseases, inflammatory conditions, and other infectious diseases should be included on the differential.^1^

Furthermore, studies have demonstrated that recent ocular surgery (cataract surgery, LASIK), solid organ transplant, and/or corticosteroid use are common predisposing factors for Nocardia infections.^1^^,^^2^^,^^9^^,^^12^ Our patient's higher susceptibility stems from her past medical history of renal transplantation on immunosuppression (including oral prednisone) and recent cataract surgery.

Immunosuppression and/or a compromised adaptive immunity were some of the consistent findings in patients who developed scleritis related to other Nocardia species.^1^^,^^12^ As a result, prompt treatment of Nocardia scleritis is crucial as a delay in proper care could lead to a detrimental impact on vision as in some cases that spread leading to endophthalmitis. However, when proper prompt treatment is initiated, the infection responds well and carries a good prognosis.^9^

The medical management for Nocardia scleritis depends on antibiotic specificity. Oral antibiotics such as TMP/SMX, ciprofloxacin, minocycline, gemifloxacin, and linezolid have shown promising results, whereas amikacin, cefazolin, and fluoroquinolone were determined to be the most effective topical antibiotics.^1^^,^^12^ However, some studies noted that ciprofloxacin might not be effective as a first-line treatment against ocular Nocardia infections.^9^ Our reported case also showed a poor response to ciprofloxacin. In brief, oral TMP/SMX and topical amikacin were considered to be the best antibiotics for the treatment of Nocardia scleritis.^1^^,^^9^^,^^12^^,^^14^ Amikacin has shown remarkable in-vitro activity against all Nocardia species.^9^ However, it is remarkable that TMP/SMX-resistant Nocardia cases have been on the rise.^11^

An interesting feature of this case included recurrent, separate conjunctival defects. It was unclear if these were directly related to *N. arthritidis*, an inherent inflammatory process related to the underlying scleritis, or related to drug toxicity. Given their uncertain nature, a biopsy was performed, and they did not represent active infection. We are not aware of other Nocardia scleritis reports documenting a similar presentation.

In addition to the correct type of antimicrobial agent, its administration route is crucial as the sclera is an avascular tissue that is highly predisposed to poor drug penetrance. Our presented case utilized several drug administration routes, including subconjunctival injections, topical antibiotics, and oral antibiotics.^15^ Interestingly, other studies have found that conservative management to treat Nocardia scleritis might not be sufficient, requiring future surgical debridement in around 75% of the cases.^1^^,^^15^^,^^16^

Despite the paucity of current literature pertaining to *N. arthritidis*-related scleritis, it appears this pathogen can be successfully treated with similar strategies used for other reported Nocardia species. Overall, Nocardia scleritis should receive prompt, aggressive treatment to avoid sight-threatening complications.

## 4 Conclusions

To our knowledge, this is a unique case report of scleritis caused by *N. arthritidis*. Nocardia scleritis is rare but may have dreadful outcomes if not treated promptly. Hence, Nocardia infection should be considered with high clinical suspicion in immunocompromised patients with recent ocular surgery, exhibiting anterior and unilateral scleritis or sclerokeratitis, accompanied by unilateral ocular pain and redness. *N. arthritidis* can be successfully treated with similar strategies used for other reported Nocardia species.

## Patient consent

The patient orally consented to publication of the case.

## Declaration of competing interest

Funding: We have no funding or grant support. Conflicts of interest: We have no relevant conflicts of interest to disclose. AYC has consulted for LayerBio. Authorship: All authors have read and agreed with the work, and have contributed in a way that justifies authorship. Acknowledgements: We would like to thank the patient for allowing us to treat his/her condition and publish it for further awareness of the diagnosis.
